# Strengthening HPV vaccination delivery: findings from a qualitative service evaluation of the adolescent girls’ HPV vaccination programme in England

**DOI:** 10.1093/pubmed/fdz061

**Published:** 2019-06-20

**Authors:** P Paterson, S Mounier-Jack, V Saliba, J Yarwood, J White, M Ramsay, T Chantler

**Affiliations:** 1 The Vaccine Confidence Project, Department of Infectious Disease Epidemiology, London School of Hygiene & Tropical Medicine, London, UK; 2 Department of Global Health and Development, London School of Hygiene & Tropical Medicine, London, UK; 3 Immunisation, Hepatitis and Blood Safety Department, Public Health England, London, UK

**Keywords:** England, qualitative research, HPV vaccine, school-based vaccination, vaccine delivery strategies

## Abstract

**Background:**

In 2014, the number of HPV vaccine doses given to adolescent girls as part of the English school-based immunization programme was reduced from three to two. This was based on evidence that a two-dose schedule provides long-lasting protection against HPV infection. In 2015/16 a small decline in HPV vaccination coverage in adolescent girls was noted; from 86.7% for the three-dose schedule in 2013/14 to 85.1% for the two-dose schedule. This evaluation examined whether service-related factors contributed to this decline.

**Methods:**

In May–August 2017, we conducted semi-structured qualitative interviews with 39 participants responsible for commissioning or delivering immunization programmes in six local authorities in the South West, North Central Midlands and South Central Midlands, England.

**Results:**

Effective planning and data management were key for successful service provision of HPV vaccination, as well as close collaboration between commissioners, service providers and data system managers, a team skill mix with experienced staff, pro-active engagement with schools and service providers equipped to respond to parental concerns.

**Conclusions:**

To maintain and improve the high HPV adolescent girls’ vaccine coverage rates achieved in England, in the context of an expanding school-based immunization programme, it is essential to strengthen the organizational capacity of the delivery system.

## Introduction

A national human papillomavirus (HPV) vaccination programme was introduced in England in 2008, with the aim of reducing the incidence of cervical cancer.^1^ Initially, the programme consisted of offering secondary school Year 8 girls (12–13 years of age) three doses of vaccine during that academic school year. HPV vaccine coverage in the academic year 2012/13 was 90.9% for at least one dose, 89.6% for two doses and 86.1% for three doses.^2^

In March 2014, the Joint Committee on Vaccination and Immunization (JCVI) recommended a change from a three to a two-dose schedule based on evidence that two doses of HPV vaccine given at least 6 months apart provided long-lasting protection against HPV infection.^2,3^ This schedule change was implemented in September 2014 using two different delivery models: (i) two doses administered in school Year 8, (ii) one dose in each of school Years 8 and 9.

The first complete HPV vaccine coverage figures for the two-dose schedule showed that there had been a slight decline in coverage in England with 85.1% of teenage girls completing the recommended two-dose course in 2015/16 compared to 86.7% of Year 9 girls completing the three-dose course in 2013/14.^4^ This was unexpected given the reduction in number of doses and increased capacity for school-based immunization delivery. Two-dose coverage rates for Year 9 girls also varied significantly across different local authorities, ranging from 43.7% to 99.1% in 2015/16.^4^ This downward trend continued in 2016/17 when 83.1% of Year 9 females completed the two-dose HPV vaccination course.^5^

A number of studies have investigated acceptability and barriers to access to HPV vaccine from the adolescents’ perspective.^6,7^ A review of organizational factors in the delivery of school-based vaccination programmes in high-income countries highlighted key factors of performance that included staff capacity and skills, and the central role of the school nurse; school leadership to support the vaccination programme; communication with parents about the purpose of vaccination and obtaining parental consent; and practical aspects of clinic organization and delivery.^8^ Few studies contrast different service delivery strategies and explore how organizational factors relate to programme performance.

The purpose of this service evaluation was to examine whether service-related factors may have contributed to a downward trend in adolescent girls’ HPV vaccination coverage and identify best practices from the perspectives of service providers and commissioners.

## Methods

### Study population, recruitment and sampling

This service evaluation was conducted in six local authorities covered by three Screening and Immunization Teams (SIT) in England: South West (Cornwall, North Somerset, Bristol), North Central Midlands (Lincolnshire, Leicester), and South Central Midlands (Luton) (Table 1). This sampling frame was determined in collaboration with PHE investigators. We included areas that; (i) delivered either the two doses of vaccine in school Year 8 and areas that delivered the first dose in Year 8 and the second dose in Year 9. (ii) were geographically and socio-demographically diverse, (iii) had a range of HPV coverage rates, and commissioned different types of providers (e.g. school nurses, and immunization teams).

**Table 1:** Study sites

Screening and Immunization Team (SIT)	Local Authority (LA) areas	Delivery model (2016/17)	Type of providers (2016/17)	Service delivery format (2016/17)	Vaccination coverage (2016/17) Year 8^9^	Vaccination coverage (2016/17) Year 9^9^
NHS England South West	**Cornwall**	Two doses over **two** academic years	Community interest company	Immunization team	Dose 1:78.6%	Dose 1:83.8%
						Dose2:57.6%
	**North Somerset**	Two doses over **two** academic years	Community interest company	Immunization team that contracts school nurse time	Dose 1:90.8%	Dose 1:91.0%
						Dose 2:83.0%
	**Bristol**	Two doses over **two** academic years	Partnership	Mixture of school and immunization nurses	Dose 1:76.4%	Dose 1:82.3%
						Dose 2:73.6%
NHS England North Central Midlands	**Lincolnshire**	Two doses over **two** academic years	Community NHS Trust	School nursing	Dose1:81.6%	Dose 1:87.5%
						Dose 2:77.7%
	**Leicester**	Two doses over **one** academic year	Community NHS Trust	Immunization team	Dose 1:84.5%	
					Dose 2:72.9%	
NHS England South Central Midlands	**Luton**	Two doses over **one** academic year	Community NHS Trust and NHS Foundation Trust	Immunization team	Dose 1:80.9%	
					Dose 2:73.9%	
**England**					Dose 1:87.2%	Dose 1:88.8%
						Dose 2:83.1%

We invited individuals working at commissioning and service delivery level (Box 1) to participate by emailing them a study information letter. Respondents who expressed interest in participating were contacted. An initial phone call was arranged for a researcher to explain the study and answer any questions. If the respondent was still interested in participating, a time and place for the interview was arranged. Researchers visited study participants at their place of work, discussed what participation would involve and obtained written informed consent prior to conducting interviews and observations. The consent process included an explanation on how we would protect participants’ confidentiality.

Box 1 Targeted participants

**Table:** 

**Commissioning level**	**Service delivery level**
NHS England Public Health Commissioners Screening and Immunization Leads Immunization managers Immunization coordinators with responsibility for school-aged immunizations	Service provider organization administrators Service provider nursing leads Nurses who provide the vaccines in schools Service provider data administrators Child Health Information Service Managers

### Data collection

This involved individual and group interviews and an observation of a school immunization session (documented in field notes). The interviews were conducted by two investigators; TC focused on the South West and Central Midlands SITs areas and PP on the North Midlands SIT area. A semi-structured interview (SSI) approach was adopted to enable the interviewer to cover pre-defined topics and allow the exchange to be shaped by interviewees’ roles, responsibilities and experiences (Box 2 and 3). The interview topic guides were pre-tested with the support of an immunization provider and a commissioner from a non-participating area. Interviews were mostly conducted face to face, or by telephone. Interviews were audio-recorded and transcribed verbatim.

Box 2 Topic areas for commissioners

**Table:** 

Types of provider (Community trust, social or private enterprise) Commissioning arrangements and contracts School-aged immunizations Process of working with providers Facilitators and barriers to commissioning school aged immunization Performance of the HPV vaccine programme across LAs/Providers Changes in the provider landscape, delivery models, data collection procedures since 2014

Box 3 Topic areas for service providers

**Table:** 

Roles and team set up Delivering the HPV vaccine programme within the context of the wider school immunization and health programme (changes in schedules, addition of new vaccines) Contextual factors and vaccine acceptability Call and recall systems, mop up strategies Contracting, submitting tenders, agreeing deliverables, communication with commissioners HPV service delivery models: delivery model A (dose1 year 8, dose 2 year 9) and delivery model B (both doses in year 8) Engagement and communication with schools, girls and their parents/guardians, and related consent procedures Data management systems and data flow (data collection, recording of vaccination status, sharing data, data quality, and communication)

### Data analysis

The transcripts were downloaded into QSR International’s NVivo 11 qualitative data analysis management software programme. PP & TC read and annotated the transcripts from their respective SIT site interviews and held regular data synthesis meetings with SMJ to develop and refine the coding framework. The approach to data analysis was thematic and involved a combination of deductive and inductive coding.^10^ This consisted of organizing the data under the pre-defined topic areas from the interview guides and then exploring this data inductively to identify the key themes and associated sub-points. The transcripts from service commissioners and providers were analysed together to explore the interactions between these actors, although where participants recounted separate experiences this was noted. Some interviewees were contacted again to address gaps in information and provide clarifications. At the final stage of analysis, the findings were discussed with co-investigators from Public Health England.

## Findings

### Study participants

Seven immunization programme commissioners and 32 service providers took part in an individual or a group SSI between May-August 2017. These exchanges lasted between 20–90 minutes. In one of the SIT areas (South West) a researcher also observed a school immunization session because the timing coincided with data collection. This was not the case at the other research sites.

### Analytical themes

Five themes were defined in relation to programme service delivery and three relating to data management. The HPV immunization programme service delivery themes are: staff skill mix, delivery teams and the role of commissioners; working with schools; information and consent logistics; immunization session logistics; and addressing concerns and negative messages. The HPV immunization programme data management themes are: accurate cohort numbers; advantages of automated and real-time database systems; and an effective data management team.

### Hpv immunization programme service delivery

#### Staff skill mix, delivery teams and the role of commissioners

Staff delivering the school-aged immunization programme varied (Table 1). Key differences between local authorities were the mix of staff (e.g. number of administrators, school/immunization nurses, immunization leads/managers) and whether vaccines were provided as part of a ‘broader school nursing service’ or by a ‘stand-alone immunization team’. Participants situated within the former noted that the competing priorities of their role had an impact on their capacity to manage the immunization programme, although they valued the direct contact with adolescents that immunization sessions afforded. Immunization teams were able to focus on providing a stand-alone service but highlighted the importance of feedback loops to the school nursing service where safeguarding concerns arose during immunization contacts with adolescents.

‘At the moment, [the HPV vaccination programme] it’s sitting within a wider programme 0-19 [years] and school nursing broadly… we’ve got immunisations to deliver, but at the same time, we’ve got emotional health and wellbeing work to do, we’ve got safeguarding work to do… which compete for the time.’ (Service provider)

‘When I’ve said, ‘Is there anything else you’d like to ask?’… they’ve said things like, ‘Yes, I’m really struggling with my sleeping at the moment’… I’d rather have the quick five-minute chat and then give them a little bit of signposting… if we’re there all day… let’s give them a positive health contact. Let’s give them the opportunity.’ (Service provider)

Planning and administration were highlighted as key to successful delivery by the majority of interviewees. Service provider teams that included designated, experienced administrators and data management staff were better placed to manage the increasing schedule of school-aged vaccines (influenza, HPV, teenage boosters and Men ACWY) and work with schools to provide these immunization programmes within the relevant schools years. Retaining experienced and suitable trained nurses and administrative staff was however also reported as a challenge by service provider leads.

Close collaboration between commissioners and providers, setting clear expectations and resolving issues together, was thought to lead to effective service delivery.

#### Working with schools: facilitating programme delivery

Specific named contacts within schools and named counterparts in immunization teams helped establish good working relationships with schools. Service providers stated that this helped promote the vaccination programme, facilitated session coordination and improved the percentage of consent forms returned, especially where schools were actively involved in chasing unreturned forms.

‘Some schools are very good and get a very good response… we tend to find it’s the ones that have got a nominated person in the school.’ (Service Provider)

#### Information and consent logistics

According to interviewees, non-returned consent forms may either not have been given to parents, or were not completed by parents due to lack of time, misplacement, or hesitancy about HPV vaccination. Some teams had adapted the PHE information and consent form template, adding vaccine and health history questions, immunization checklists and space for post vaccination observations.

‘One of the biggest issues, is not getting the forms returned. So, it’s not actually a positive refusal, but it’s not a positive consent either.’ (Service provider)

The logistics of forms passing through multiple ‘hands’ was an evident barrier and meant that students would sometimes turn up to immunization sessions without a completed consent form. Some nurses would confirm if the student wanted to be immunized and try to contact the parent to obtain permission to administer the HPV vaccine. This could be time-consuming and some areas were planning to maximize form return by using e-consent systems or streamlining consent form dissemination (e.g. using school internet portals).

#### Immunization session logistics

The observation revealed that sessions could be very busy, with students lining up outside rooms where nurses set up stations while trying to use screens to maintain individual’s privacy. Nurses highlighted in interviews that the management of these sessions benefitted greatly from active involvement of key school staff helping coordinate the arrival of students and accompanying individuals when necessary. Some schools also offered the option of using a first aid room for students with special needs or who needed closer attention or counselling. Nurses reported that they tried their best to provide ‘adolescent-centred care’ and take note of health issues of concern either raised by students or observed.

‘If we have young people that… are prone to feeling faint, prone to feeling unwell… [we] say, ‘Come and see us when… it’s a bit quieter and then we can give you a bit more time… if the schools can identify them to us, maybe we could administer their vaccine in… the first aid room and do it with more privacy.’ (Service provider)

#### Addressing concerns and negative messages

In December 2016, a UK-based day time television interview with Melinda Messenger, a presenter and former glamour model, sparked social media discussion about the safety of the HPV vaccine.^11^ A few interviewees stated that this had resulted in some vaccine hesitancy amongst parents. Although not presented as a major challenge, interviewees highlighted the need for nurses to be prepared (receive regular training and have sufficient access to informational materials) to pre-empt, acknowledge and address parents’ concerns whilst providing information about the benefits and risks of the vaccine. Several participants noted the importance of these discussions.

‘I have had a few [parents] that have been thinking they’re going to say no [to HPV vaccine], but then we’ve had a conversation and it’s actually allayed their fears and… they actually go ‘okay, yes, we’ll have it.’’ (Service provider)

### HPV immunization programme data management

#### Accurate cohort numbers

Interviewees stated that it was key to obtain accurate class lists in advance (ideally before the start of the new school year) of the vaccination programme starting, for the team to effectively plan vaccination activities.

‘To get your cohort numbers correct in the beginning, is the key to starting a good programme basically… you’re not going to get your coverage right, are you, unless you actually have got your denominator right in the beginning.’ (Service provider)

The migration of adolescent girls between schools and geographies was reported as difficult to manage, since it could result in class list inaccuracies, incorrect numerators or denominators and inaccurate vaccination coverage statistics. Any movement in the time span between the first and second dose of the HPV vaccine made it particularly difficult to trace and ensure adolescent girls were fully protected against HPV infection. Service providers who used a one year delivery model (two HPV vaccines in school Year 8) noted that there was less movement of children between schools or areas. Real-time database systems also helped manage this, as did troubleshooting meetings between commissioners, Child Health Information Services (CHIS) leads and service providers, and regular communication with General Practice.

#### Advantages of automated and real-time database systems

Inputting and cleaning data in database systems was highlighted as labour intensive, especially the parts of the data management system that are not automated.

‘There’s a lot of matching going on and it’s all a very manual process… 18 000 mismatches that needed to be sorted manually and some of them used to take 20 minutes each.’ (Service provider)

Database systems that are automated, with the ability to conduct bulk processing, increase efficiency and accuracy. When database systems are not real-time, delays between records appearing on GP or school provider servers lead to inaccurate data monitoring and could result in dual immunization. For those on the CHIS system, this can also happen when GPs do not send updated vaccination records to CHIS in a timely fashion.

‘They both said I think we’ve had this done at the GP surgery. So, we rang the GP surgery and they have had it done but that information wasn’t on CHIS.’ (Service provider)

#### An effective data management team

The effectiveness of the data management team was reported to be reliant on regular training, up-to-date system operating practices and effective support from the database system and the software teams. If the data management team were located together it was easier to implement updates and changes to data management. Having dedicated administrative staff within teams was also viewed as key to effective programme delivery, as were good working relationships within the CHIS team, and between the CHIS and the immunization team.

‘Teams are scattered over four bases so it’s not as easy to make very sudden quick changes to processes, and then ensure that that’s communicated.’ (Service provider)

## Discussion

### Main findings of this study

This study sought to determine service-related factors that may have contributed to the slight downward trend in HPV vaccination coverage since 2014 and identify ‘good practice’ which could mitigate these.

Systematic planning and accurate data management systems was viewed as key for the successful service provision of HPV vaccination, as well as close collaboration between commissioners, service providers and data system managers, pro-active engagement with schools, and service providers being equipped to respond to parental concerns. Organizational and logistical aspects (e.g. skill-mix, obtaining consent, administrative capacity in schools, data management) of school-based vaccination were identified as factors which could influence vaccination coverage.

### What is already known on this topic

In their review, Perman and colleagues identified the influence of programme leadership and governance, workforce capacity, communication with parents and students, and clinic organization on the effective delivery of vaccination programmes in school-based settings.^8^ School staff commitment and the quality of the relationship with a school nurse was also highlighted as key for high uptake of HPV vaccination^12^ while delays in getting accurate and up-to-date class lists was noted as an impediment.^13^ Lessons learned from English service providers highlighted follow-up and reminders for girls who missed immunization sessions and the provision of additional opportunities for HPV vaccination as factors associated with high coverage.^5^ One study has shown preliminary evidence that incentivising vaccination in school may improve vaccination uptake.^14^

Several studies have reported that school nurses felt burdened by the workload associated with school vaccination programmes^15,16^ while staff absenteeism and reduction in school nursing capacity are associated with lower performance.^5,15^ In addition, specific challenges linked to the collection and interpretation of data have been reported by UK vaccination providers. These include possible underestimation of coverage due to incomplete accounting of ‘mop-ups’, movement of students in and out of schools affecting the numerators and/or denominators, unaccounted vaccination at GP practices, the difficulty of combining data from multiple sources, and issues of inadequate data quality.^5,17^

### What this study adds

Findings from this study were consistent with previously cited factors of performance of school vaccination programmes. Our evidence emphasizes the increasing importance of organizational delivery to the success of an expanded vaccination schedule. It also highlights good practices for both providers and commissioners.

These include the need to engage with schools for the long haul and the allocation of adequate administrative resource to support effective interactions between immunization teams, the school nursing service and schools. Service providers also suggested ensuring that staff have sufficient experience and a good skill mix including planning, data management, administrative and clinical skills for successful service provision of HPV vaccination. Close collaboration between commissioners, service providers, data system managers and schools is needed to ensure that providers are well equipped to respond to parental concerns. Finally, particular attention was placed by service commissioners and providers on developing and testing effective way to manage consent of adolescents in school programmes.

### Limitations of this study

This study aimed to explore the views and perspectives of service commissioners and providers to identify factors contributing to high- and under-performance of school-based HPV vaccination. Although our sample was limited to three SITs, care was taken to select geographical areas where; i. differences in vaccine coverage across local authorities existed and ii different delivery models were being used, in terms of schedule (2 HPV doses given in one or two school years) and organizational delivery (school nursing service or standalone immunization teams). Obtaining data from commissioners, providers and data managers enabled us to triangulate information and obtain a rich analysis of factors affecting all levels of service delivery. We acknowledge that interview participants may have been affected by social desirability bias, giving responses expected to please the interviewers, although the use of a topic guide helped mitigate against this. Conducting more than one observation of school immunization sessions would also have strengthened our analysis.

## Conclusions

England has achieved high coverage rates for HPV vaccine compared with other European countries, and can be considered a front-runner among high-income countries for school-based delivery of vaccines, given its expanded schedule and strong performance. However, the slight decrease in coverage for HPV vaccine coverage since 2014 combined with interviewees’ reported views of the complexity of organizational and logistical aspects of delivery may reflect a growing imbalance between workload capacity and/or a delivery model that needs to be adapted to its new expanded scope. Learning from this rapid expansion of the school vaccination schedule should be of interest to other high-income countries, and constitute an opportunity for public health authorities to further improve the design of these vaccination programmes.
